# Disseminated Cryptococcosis in a Non-HIV Patient in Singapore

**DOI:** 10.1155/2019/3835701

**Published:** 2019-09-22

**Authors:** Jonathan See, Kok Choon Raymond Fong, Humaira Shafi

**Affiliations:** Department of Infectious Disease, Changi General Hospital, 2 Simei Street 3, Singapore 529889

## Abstract

We present a case of disseminated cryptococcosis (DC) in a 71-year-old gentleman with systemic lupus erythematosus (SLE) on long-term corticosteroids. He initially presented with right arm cellulitis in a tertiary hospital in Singapore and was subsequently diagnosed with DC involving skin, meninges, blood, and possibly pulmonary involvement. He eventually succumbed to the disease despite prolonged antifungal therapy. Through this case, we wish to highlight an atypical clinical presentation of an uncommon infection and hope to share the importance of considering DC in the differential diagnosis of nonresolving cellulitis among immunocompromised individuals. Mortality and morbidity rates for this condition remain high despite appropriate treatment. Early diagnosis and treatment are crucial for improved outcomes. More research is needed to improve the therapeutic modalities for treatment of DC and to improve the clinical outcomes for this life-threatening condition.

## 1. Introduction

Cryptococcosis is a disease caused by a basidiomycetous encapsulated yeast-like fungal organism [1]. Two pathogenic species of cryptococcus are known to cause disease in humans. *Cryptococcus gattii* is most commonly responsible for cryptococcosis in immunocompetent individuals, while *Cryptococcus neoformans* (CN) being more common in immunocompromised patients, which include HIV-positive patients, patients with systemic lupus erythematosus (SLE), patients on prolonged immunosuppression therapy, and transplant recipients [1–3].

*Cryptococcus neoformans* can be found in the natural environment and is often associated with dust, soil, contaminated food, plants, and bird faeces [1, 2]. Clinical manifestation can range from asymptomatic pulmonary colonisation to life-threatening meningitis and disseminated infection [4, 5] with pulmonary and central nervous system (CNS) being the most common sites reported for patients with disseminated cryptococcosis (DC) [3, 6]. Skin involvement is generally rare in cryptococcosis, and it occurs in 10–20% of patients. Cutaneous cryptococcosis typically presents with different clinical morphologies including acneiform papules, ulcers, subcutaneous nodules, and, rarely, as cellulitis [7, 8]. Cryptococcal cellulitis, in particular, is usually indistinguishable from bacterial forms, and its delayed diagnosis usually predicts a worse outcome [7, 9, 10].

In Southeast Asia, cryptococcosis is common amongst HIV-infected individuals, with an estimated 120 cases per 1000 HIV-infected individuals per year [11]. SLE patients represent an important group who are susceptible to infection because of disease-related immunological defects and concurrent immunosuppression [12]. The growing size of the immunocompromised patient population from chemotherapy and biological agents further contributes to the medical importance of cryptococcosis [13].

## 2. Case Presentation

A 71-year-old male patient with a past medical history of SLE and lupus nephritis, who had been on prednisolone for 20 years, was referred to our hospital by his general practitioner after presenting with right arm swelling and erythema of 5 days duration that did not improve despite oral amoxicillin/clavulinic acid. A review of system was unremarkable for headaches, confusion, cough, sputum, shortness of breath, and fever. He had not been exposed to any bird excrement.

**Figure 1:**
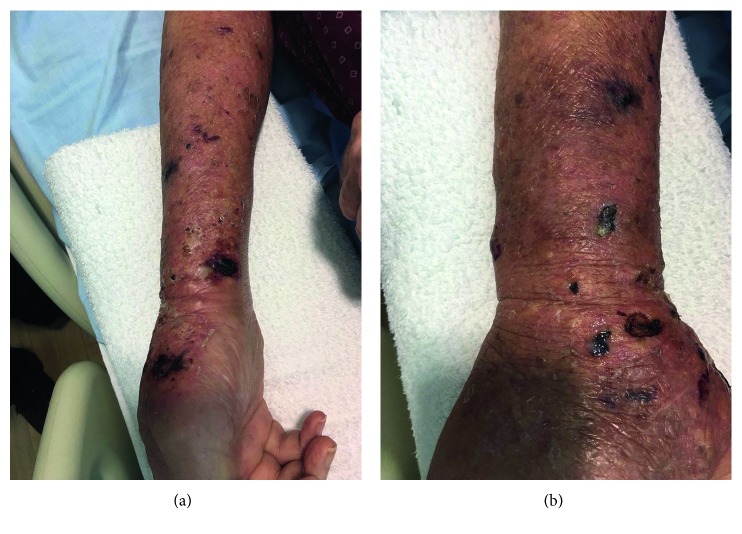
Patient's right forearm cellulitic skin changes and bullae on admission.

On admission he was afebrile, his blood pressure was 122/88 mmHg, pulse rate was 82/min, and respiratory rate was 18/min and was saturating at 98% on room air. He was noted to have left basal crepitation and bipedal edema. Skin examination (Figure 1) showed diffuse right upper limb pitting edema and erythema, with multiple 1-2 cm hemorrhagic tender bullae scattered over the forearm.

On day 1 of admission, blood cultures were taken, investigations were done (Table 1), and intravenous (IV) cefazolin was started. On day 3, antibiotics were escalated to IV piperacillin-tazobactam because of the temperature of 37.7°C, persistent swelling, and erythema.

Blood culture yielded CN on day 6 of admission and amphotericin-B was commenced. Lumbar puncture was performed which isolated CN on cultures. Antifungal therapy was optimized to liposomal IV amphotericin-B (AmBisome based on 4-5 mg/kg) and oral flucytosine 1 g Q12H (based on 25 mg/kg).

**Figure 2:**
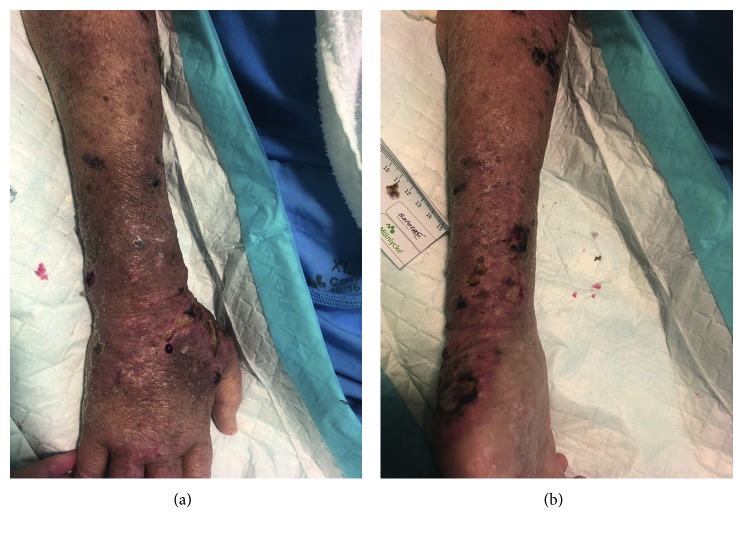
Patient's right forearm improvement 10 days after appropriate antifungal therapy.

After further investigations (Table 2), clinical impression was for DC involving blood, CNS, lung, and skin involvement. Modest improvement in the skin infection was noted after starting antifungal therapy (Figure 2). Biochemically, CRP also downtrended (Table 3).

Blood culture was sterilised after 8 days of starting antifungals, but CSF culture remained positive for CN at day 10 of treatment (Table 4).

Prednisolone 15 mg/day was lowered to 10 mg/day after discussion with rheumatologist. Further hospital course was complicated by acute on chronic thrombocytopenia secondary to flucytosine, which led to discontinuation of flucytosine after 19 days of administration. He also developed worsening of chronic kidney disease (CKD), fluid overload, haemorrhoidal bleeding, and worsening of anemia. AmBisome was discontinued after 22 days, and the patient was terminally discharged to home in keeping with his wishes. He demised 30 days after initial presentation.

## 3. Discussion

Cryptococcosis is a serious infection that predominantly affects immunocompromised patients. In this case, the patient was on long-term corticosteroids for 20 years for SLE. He did not receive disease modifying agents. Our patient also had CKD-3 secondary to lupus nephritis. As reported in multiple studies, patients with higher mortality risk in cryptococcosis have baseline renal impairment, disseminated disease, and HIV-negative; all 3 factors are present for our patient [14–16].

Cryptococcosis presenting with skin infection/cellulitis is atypical. International literature has shown that skin involvement occurs in only 10–20% of patients [7, 8]. In a large retrospective Singapore study in 2014, only 6 out of 62 patients (9.7%) with cryptococcosis had skin findings [17, 18]. Presenting symptoms were fever (79%), headache (71%), and cough (45%), all of which were absent in our patient during initial presentation. Furthermore, cryptococcosis occurring in HIV-negative patients is relatively rare as well; only 19% of patients (12 out of 62) in the same study reported cryptococcosis in HIV-negative.

An atypical presentation coupled with atypical risk-group contributed to a delayed diagnosis in our reported case. This corresponds to the study by Chan et al. which revealed that delay in treatment significantly increased in HIV-negative patients (*p*=0.03) [17, 18]. Prognosis of non-HIV patients with DC is relatively poor compared to HIV patients; studies have reported 1-month mortality rates of 63%, with 81% of deaths occurring within 2 weeks of diagnosis [16]. Delayed diagnosis leading to delay in appropriate treatment is likely to contribute to increased mortality in non-HIV patients.

This case highlights several important points. Firstly, clinicians need to recognise cryptococcosis as a severe systemic infection as it can yield unfavorable outcomes despite treatment, more so in the setting of late diagnosis and initiation of treatment. Secondly, inflammatory skin changes should be promptly investigated for differential diagnoses other than bacterial in immunocompromised hosts and when not responding to conventional antimicrobial treatment as in our case. Thirdly, this case also illustrates how cryptococcosis is known to masquerade conditions like bacterial cellulitis, panniculitis, papules, pustules, nodules, abscesses, edema, ulcers, and mollusum contagiousum-like lesions [19]. Such a wide range of skin presentations makes clinching the diagnosis challenging; in our case, initial dermatology consult suspected the presentation to be due to edema blisters. The need to consider a broader differential diagnosis early in the setting of atypical skin infections in the immunocompromised population cannot be overemphasized through this case.

Lastly, this case also highlights the present challenges with treatment of cryptococcosis. Current recommendations for anticryptoccocal treatment include amphotericin-B (L-AMB as a substitute for amphotericin-B in cases of intolerance) with flucytosine, in non-HIV, nontransplant hosts [20]. Antifungals have side effects and complications, including hematological cell line suppression and renal toxicity. This may prevent efficacious antifungal treatment in patients with comorbid conditions. Furthermore, in autoimmune conditions, antifungal treatment can be further complicated by the complexity of the primary disease. Reduction in immunosuppression has to be balanced against risk of flare, leaving physicians with a delicate balance to thread upon. This is in contrast with HIV patients with cryptococcosis, whereby stronger antiretroviral therapy only serves to aid in the management of both cryptococcosis and HIV, allowing physicians with less management dilemma.

## Figures and Tables

**Table 1:** Investigations on day 1 of admission.

Test	Findings
C-reactive protein (CRP)	112.6 mg/L
Procalcitonin	0.44 *μ*g/L
White blood cell count	5.3 × 10^9^/L
Neutrophil count	4.5 × 10^9^/L
Lymphocyte count	0.3 × 10^9^/L
HIV screen	Nonreactive
Chest X-ray	No confluent consolidation is detected. Linear atelectasis of the left lower zone is noted with a stable left pleural effusion

**Table 2:** Further investigations (day 7–10) leading to diagnosis of disseminated cryptococcosis.

Test	Findings
Serum cryptococcal antigen (CRAG)	Titer ≥ 1 : 2560
Blood fungal culture	CN
Computed tomography (CT) thorax scan	Patchy areas of consolidation in the left perihilar region and right lower lobe along with scattered subcentimeter pulmonary nodules which suggested infective changes of pulmonary cryptococcosis on a background of fluid overload contributed by congestive cardiac failure and worsening renal function
CT brain scan	No space occupying lesion, no mass effect, and no intracranial hemorrhage or territorial infarcts
Lumbar puncture	
Opening pressure	10.0 cm water
Cerebrospinal fluid (CSF) gram stain	Encapsulated blasticonidia (yeasts)
CSF fungal microscopy	Encapsulated blasticonidia seen, morphology suggestive of *Cryptococcus*
CSF CRAG	Titer ≥ 1 : 2560
CSF fungal culture	CN
CSF aerobic culture	CN
Skin swab taken from blisters	
Skin fungal culture	CN
Skin aerobic culture	CN

**Table 3:** C-reactive protein trend in response to antifungal therapy.

CRP (mg/L) on day 6	CRP (mg/L) on day 10	CRP (mg/L) on day 14
117.2	41.7	33.6

**Table 4:** Repeated microbiological investigations after 10 days of treatment.

Test	Findings
Serum CRAG	Titer ≥ 1 : 2560
Blood fungal culture	Negative after 8 days of starting combination antifungals
Lumbar puncture	
Opening pressure	15.0 cm water
CSF gram stain	Encapsulated blasticonidia (yeasts)
CSF fungal microscopy	Encapsulated blasticonidia seen, morphology suggestive of *Cryptococcus*
CSF CRAG	Titer ≥ 1 : 2560
CSF fungal culture	CN
CSF aerobic culture	CN

## Additional Points

*Learning Points*. (1) Cryptococcal and atypical mycobacterial skin infections should be reasoned in immunocompromised patients including non-HIV patients because delay in diagnosis and treatment corresponds with increased mortality and morbidity. Skin manifestation of DC, while rare, should be considered when lack of response is observed with conventional antimicrobial therapy for skin and soft tissue infections. (2) Recognising the difficulties in management of cryptococcosis with concurrent autoimmune conditions—multidisciplinary teams may be consulted early to strike a fine balance between adequate immunosuppression and promoting a stronger immune response against the ongoing infection. (3) Mortality remains substantial. Antifungal treatment for cryptococcosis—despite extensive knowledge of cryptococcosis in recent years, our treatment modalities remain limited. Serious side effects limit the efficacy of treatment. Safer and potent antifungal treatment modalities are needed with viable second-line and even third-line alternatives to fall back upon, should first-line treatment be contraindicated due to side effects.

## References

[1] Probst C., Pongratz G., Capellino S. (2010). Cryptococcosis mimicking cutaneous cellulitis in a patient suffering from rheumatoid arthritis:a case report. *BMC Infectious Diseases*.

[2] Srivastava G. N., Tilak R., Yadav J., Bansal M. (2015). Cutaneous cryptococcus: marker for disseminated infection. *BMJ Case Reports*.

[3] Valente E. S., Lazzarin M. C., Koech B. L. (2015). Disseminated cryptococcosis presenting as cutaneous cellulitis in an adolescent with systemic lupus erythematosus. *Infectious Disease Reports*.

[4] Perfect J. R., Durack D. T., Gallis H. A. (1983). Cryptococcemia. *Medicine*.

[5] Levitz S. M. (1991). The ecology of Cryptococcus neoformans and the epidemiology of cryptococcosis. *Clinical Infectious Diseases*.

[6] Singh N., Dromer F., Perfect J. R., Lortholary O. (2008). Immunocompromised hosts: cryptococcosis in solid organ transplant recipients: current state of the science. *Clinical Infectious Diseases*.

[7] Hall J. C., Brewer J. H., Crouch T. T., Watson K. R. (1987). Cryptococcal cellulitis with multiple sites of involvement. *Journal of the American Academy of Dermatology*.

[8] Chaya R., Padmanabhan S., Anandaswamy V., Moin A. (2013). Disseminated cryptococcosis presenting as cellulitis in a renal transplant recipient. *The Journal of Infection in Developing Countries*.

[9] Lu H.-C., Yang Y.-Y., Huang Y.-L. (2007). Disseminated cryptococcosis initially presenting as cellulitis in a rheumatoid arthritis patient. *Journal of the Chinese Medical Association*.

[10] Ni W., Huang Q., Cui J. (2013). Disseminated cryptococcosis initially presenting as cellulitis in a patient suffering from nephrotic syndrome. *BMC Nephrology*.

[11] Park B. J., Wannemuehler K. A., Marston B. J., Govender N., Pappas P. G., Chiller T. M. (2009). Estimation of the current global burden of cryptococcal meningitis among persons living with HIV/AIDS. *AIDS*.

[12] Caza T., Oaks Z., Perl A. (2014). Interplay of infections, autoimmunity, and immunosuppression in systemic lupus erythematosus. *International Reviews of Immunology*.

[13] Pappas P. G., Perfect J. R., Cloud G. A. (2001). Cryptococcosis in human immunodeficiency virus-negative patients in the era of effective azole therapy. *Clinical Infectious Diseases*.

[14] Galanis E., Macdougall L., Kidd S., Morshed M., British Columbia Cryptococcus gattii Working Group (2010). Epidemiology of Cryptococcus gattii, British Columbia, Canada, 1999–2007. *Emerging Infectious Diseases*.

[15] Seaton R. A., Naraqi S., Wembri J. P., Warrell D. A. (1996). Predictors of outcome in Cryptococcus neoformans var. gattii meningitis. *QJM*.

[16] Chuang Y.-M., Ho Y.-C., Chang H.-T., Yu C.-J., Yang P.-C., Hsueh P.-R. (2008). Disseminated cryptococcosis in HIV-uninfected patients. *European Journal of Clinical Microbiology and Infectious Diseases*.

[17] Chan M., Lye D., Mar K. W., Chow A., Barkham T. (2014). Clinical and microbiological characteristics of cryptococcosis in Singapore: predominance of Cryptococcus neoformans compared with Cryptococcus gattii. *International Journal of Infectious Diseases*.

[18] Speed B., Dunt D. (1995). Clinical and host differences between infections with the two varieties of Cryptococcus neoformans. *Clinical Infectious Diseases*.

[19] Kikuchi N., Hiraiwa T., Ishikawa M. (2016). Cutaneous cryptococcosis mimicking pyoderma gangrenosum: a report of four cases. *Acta Dermato Venereologica*.

[20] Perfect J. R., Dismukes W. E., Dromer F. (2010). Clinical practice guidelines for the management of Cryptococcal disease: 2010 update by the Infectious Diseases Society of America. *Clinical Infectious Diseases*.

